# Mental health amidst multiple crises: trends and sociodemographic risk factors in Austria’s general population

**DOI:** 10.3389/fpsyt.2025.1534994

**Published:** 2025-02-27

**Authors:** Elke Humer, Christoph Pieh, Thomas Probst, Carina Dinhof, Yvonne Schaffler, Marina Zeldovich

**Affiliations:** ^1^ Department for Psychosomatic Medicine and Psychotherapy, University for Continuing Education, Krems, Austria; ^2^ Faculty for Psychotherapy Science, Sigmund Freud University Vienna, Vienna, Austria; ^3^ Division of Psychotherapy, Department of Psychology, Paris Lodron University Salzburg, Salzburg, Austria; ^4^ Institute of Psychology, University of Innsbruck, Innsbruck, Austria

**Keywords:** depression, anxiety, insomnia, alcohol misuse, stress, pandemic aftermath, socioeconomic factors, inflation

## Abstract

**Background:**

In recent years, mental health in Austria has faced substantial challenges due to a series of both global and regional crises.

**Objective:**

This study aimed to assess changes in mental health indicators within the Austrian population over time from April 2022 to October 2024 and to identify sociodemographic correlates of poor mental health.

**Methods:**

Two cross-sectional online surveys on representative samples of the Austrian general population were conducted at two timepoints: April 2022 (n = 1,032) and October 2024 (n = 2,025). Mental health indicators, including depression, anxiety, insomnia, alcohol misuse, and stress, were measured using validated scales. Sociodemographic data were collected to examine associations with mental health indicators. Chi-squared tests and t-tests were conducted to compare mental health indicators between 2022 and 2024, and multivariable logistic regression models were applied to examine associations with sociodemographic data.

**Results:**

Clinically relevant symptoms of depression and high stress decreased from 2022 to 2024 (depression from 28.3% to 21.6%, stress from 56.3% to 51.0%; p < 0.01), while other symptoms remained stable. Sociodemographic analysis revealed that female gender, younger age, lower income, unemployment, migration background and lack of partnership were associated with higher odds for several mental health issues.

**Conclusions:**

Findings suggest small improvements in mental health from 2022 to 2024 and identified sociodemographic risk factors linked to mental health vulnerabilities.

## 1 Introduction

The prevalence of mental health issues has increased in recent years, becoming an urgent public health priority across Europe and beyond (1). Studies indicate that these challenges were exacerbated by the COVID-19 pandemic, which severely disrupted daily life and intensified pre-existing social and economic challenges (2).

In Austria the COVID-19 pandemic led to a strict lockdown during the onset of the pandemic in 2020, followed by a series of nationwide and regional lockdowns from November 2020 to May 2021, as new variants fueled waves of infection. By late 2021 the Delta variant led to increased infection rates impacting critical infrastructure and leading to high ICU occupancy. Austria introduced a controversial nationwide lockdown that was extended until January 2022 exclusively for unvaccinated individuals, alongside one of Europe’s earliest vaccination mandates, which took effect briefly in early 2022 before being suspended in July 2022. The milder course of the Omicron variant allowed easing of measures in spring 2022. In April 2022, quarantine rules were relaxed, and mask mandates were lifted in most settings by summer, except healthcare facilities. By the end of 2022, COVID-19 had largely receded from public and political focus as infection rates stabilized (3).

During the periods of lockdowns, quarantine, and social distancing, feelings of loneliness and anxiety increased in many countries, particularly among individuals with pre-existing mental health conditions (2, 4). Surveys representative for the general Austrian population conducted in April 2020—after the first four weeks of lockdown—revealed higher mental health symptoms (21% depression, 19% anxiety, 16% insomnia) compared to pre-pandemic data (5). Research from April 2022, the post-peak phase of the pandemic, found that individuals continued to report elevated levels of anxiety and stress and even higher rates of depressive symptoms (28%) compared to April 2020, indicating a sustained impact on mental well-being (6). This prolonged psychological burden might be partly attributed to the residual effects of the pandemic, with lingering uncertainties about health, social stability, and future crises.

Alongside the pandemic, other global and regional crises impacted the Austrian population. Geopolitical tensions, such as the war in Ukraine, have had widespread repercussions, affecting several aspects of life, from energy prices to migration patterns. These disruptions likely still intensify the strain on mental health, as rising living costs and economic uncertainty added new layers of stress (7).

Austria’s economic trajectory from 2020 to 2024 shows significant fluctuations, influenced by the COVID-19 pandemic and the war in Ukraine. 2020 GDP dropped sharply by 6.3%, but the economy rebounded with growth rates of 4.8% in 2021 and 5.3% in 2022 before declining again in 2023 and 2024 (-1.0% and -0.6%, respectively) (8). Inflation surged to unprecedented levels, reaching 8.6% in 2022—the highest since the 1974 oil crisis, which saw 9.5% inflation. This spike was driven primarily by the war in Ukraine, which led to a rapid rise in energy costs, with household energy prices increasing by 37%, fuel by 42%, and food by 11%. By October 2024, inflation stabilized at 1.8% (9). Austria’s unemployment rate rose sharply to 9.9% in 2020 due to the pandemic, remaining elevated at 8.0% in 2021. By 2022, it declined below pre-pandemic levels to 6.3%. In late 2024, the unemployment rate was estimated at 6.6% (8). Real incomes saw minimal change in 2020 (+0.8%) and 2021 (-0.1%) but dropped significantly by 3.4% in 2022 and an additional 0.9% in 2023. A positive shift occurred in 2024, with real incomes rising by 4.6% (8).

In addition to the pandemic, the war in Ukraine, economic instability, and other global and regional challenges might have placed further strain on the Austrian population. In October 2023, the Middle East conflict escalated, further intensified in autumn 2024. Moreover, climate change continued to have an immediate impact. The summer of 2024 marks Austria’s fourth consecutive season of extreme warmth (10). The preceding seasons—the autumn of 2023, the winter of 2023/24, and the spring of 2024—also ranked among the top three warmest on record for their respective periods (10). The summer of 2024 was followed by a massive flooding event in September 2024, combined with storms and snowfall in the high mountains. Lower Austria and Vienna experienced the most significant impact from extreme regional rainfall (11).

For Austrians, these overlapping crises create a backdrop that may worsen existing mental health symptoms or give rise to new ones. Ongoing exposure could also lead to habituation and increased resilience, potentially lowering the perceived severity of threats. The cumulative impact of the various challenges on mental health remains unclear, particularly in terms of how certain demographic groups may exhibit greater vulnerability or resilience.

Mental health is influenced by a range of sociodemographic factors, which can determine access to resources, social support, and opportunities for coping. Migration background, for example, is associated with unique stressors, including acculturative stress, discrimination, and, in some cases, economic hardship (12). Refugees, who represent a growing segment of Austria’s migrant population due to ongoing crises in the Middle East, face compounded mental health challenges. These challenges often stem from pre-migration trauma and are intensified by the complexities of resettlement. As a result, mental health disorders remain notably common among war refugees even years after resettlement (13). Education, employment, and income are also well-established correlates of mental health. Lower educational attainment is often linked to reduced health literacy, fewer employment opportunities, and lower income levels (14), which can, in turn, increase stress and vulnerability to mental health issues. Economic strain due to low income or employment instability is a potent risk factor, as it can lead to a chronic state of insecurity, contributing to higher levels of mental health symptoms (15). Partnership status is an additional factor with complex relationships to mental health. While stable relationships can provide emotional support, relationship strain can contribute to stress and depressive symptoms (16, 17).

This study aims to investigate the prevalence of mental health symptoms within the Austrian general population over two critical time points: April 2022 and October 2024. By comparing these time points, we aim to explore how mental health has evolved from the post-peak phase of the pandemic to a period marked by other compounding crises.

Additionally, we aim to identify sociodemographic correlates, including migration background, education, income, partnership status, and employment, that may be associated with clinical levels of mental health symptoms in the 2024 sample. These analyses are essential for understanding how various population segments experience mental health challenges differently, providing a foundation for targeted interventions and public health planning.

## 2 Methods

### 2.1 Study design

Data were collected via two cross-sectional online surveys conducted on representative samples of the Austrian general population. The primary survey was administered from October 10 to October 28, 2024, and was administered through Marketagent.com online research GmbH (Baden, Austria). Participants had to be at least 14 years old, reside in Austria and have internet access. Respondents were selected using quota sampling for the key demographics of age, gender, age × gender, region, and educational level. A total of 55,069 people were contacted, with 2,836 starting the survey. Of these, 139 participants discontinued the survey, and 81 people were excluded based on not fulfilling age and residence criteria. Further 591 participants who exhibited unusual response patterns, such as exceptionally fast completion times, were excluded. Those who met the participation criteria (regarding age and residence) and completed the survey received an incentive of 110 bonus points, which were credited to their Marketagent account as panel members.

The sociodemographic characteristics of the study sample (N = 2,025) are summarized in **Supplementary Table 1**. Due to low response numbers, gender-diverse individuals (n = 4) were excluded and the category “no schooling” (n = 3) was combined with “secondary school” for statistical analyses.

For comparative analysis, data from an earlier survey conducted between April 19 and April 26, 2022, were utilized. This previous survey also recruited a representative sample of the Austrian population (N = 1,032) and used identical measures to assess mental health outcomes of depression, anxiety, insomnia, alcohol misuse and stress. Findings from the 2022 survey are already published (6), and the data serve here exclusively to provide a temporal comparison of mental health trends in Austria.

### 2.2 Questionnaires

Eight key sociodemographic variables were collected: gender (female, male, diverse), age (years), region (Vienna, Upper Austria, Lower Austria, Carinthia, Styria, Tyrol, Salzburg, Burgenland, Vorarlberg), highest education level (none; secondary school; apprenticeship; vocational secondary school; higher secondary school; university), monthly net household income (<€1,000; €1,001–€2,000; €2,001–€3,000; €3,001–€4,000; >€4,000), employment status (employed; unemployed; retired), migration background (whether the participant or both parents were born outside Austria), and relationship status (single; in a partnership).

Depression symptoms were measured using the Patient Health Questionnaire (PHQ-9) (18). This nine-item self-report scale has a four-point scale (0 = not at all to 3 = nearly every day), resulting in scores from 0 to 27. Cut-offs for clinically relevant depressive symptoms were set at ≥10 for adults and ≥11 for participants aged 14-17. Cronbach’s alpha in this sample was 0.89.

Anxiety symptoms were evaluated using Generalized Anxiety Disorder scale (GAD-7) (19). This seven-item measure also uses a four-point scale (0 = not at all to 3 = nearly every day), with scores ranging from 0 to 21. Clinically relevant anxiety was indicated by cut-offs of ≥11 for adolescents and ≥10 for adults. Cronbach’s alpha was 0.91.

The Insomnia Severity Index (ISI-7) assessed sleep quality and insomnia symptoms through seven self-rated items on a five-point scale (0–4) (20). Total scores range from 0 to 28, with scores of 15 or above signifying clinically relevant insomnia. Cronbach’s alpha in this sample was 0.86.

Alcohol misuse was screened using the CAGE questionnaire, consisting of four yes/no questions (21). A score of 2 or more suggests potential clinically relevant alcohol abuse or dependence. Cronbach’s alpha was 0.66 in this study.

Perceived stress was evaluated with the Perceived Stress Scale (PSS-4) (22), which scores responses on a five-point scale (0 = never to 4 = very often). The total score ranges from 0 to 16, with a score of 6 or higher indicating high stress. Cronbach’s alpha was 0.75.

### 2.3 Statistical analyses

Chi-squared tests were used to examine differences in the prevalence of clinically relevant symptoms of depression, anxiety, insomnia, alcohol abuse, and stress between the 2022 and 2024 surveys. T-tests for independent samples were applied to examine differences in mean symptom scores of the PHQ-9, GAD-7, ISI-7, CAGE and PSS-4 between both survey periods. All tests were two-sided, with a significance level set at p < 0.05. Additionally, Cohen’s d was calculated to assess the effect size for differences in mean symptom scores.

Associations of mental health indicators with sociodemographic variables in 2024 were evaluated using binary logistic regression analyses. To account for the interrelationships between sociodemographic factors—such as the association between income and employment situation or the higher proportion of migrants in Vienna compared to other Austrian regions— multivariable logistic regression was applied to assess the independent effect of each sociodemographic variable on the prevalence of clinically relevant mental health symptoms. All assessed sociodemographic variables (gender, age, migration background, region, partnership status, employment status, income, and education) were included as predictors in the multiple linear regression model. The mental health outcomes (clinically relevant symptoms of depression, anxiety, insomnia, alcohol abuse, and stress) served as dependent variables. Adjusted odds ratios (aORs) and 95% confidence intervals (CIs) were calculated.

Analyses were conducted using SPSS version 26 (IBM Corp, Armonk, NY, USA).

## 3 Results

### 3.1 Mental health indicators in October 2024 vs. April 2022

As summarized in **Table 1**, symptoms of depression decreased from 28.3% to 21.6% from 2024 to 2022 (p <0.001). No differences were observed for symptoms of anxiety (16.2% in 2022 vs. 14.8% in 2024; p = 0.33), insomnia (14.5% in 2022 vs. 14.2% in 2024; p = 0.77), and alcohol abuse (17.9% in 2022 vs. 20.9% in 2024; p = 0.055). Symptoms of high stress decreased from 56.3% to 51.0% from 2024 to 2022 (p = 0.006).

**Table 1:** Proportion of participants exceeding the cut-off scores for symptoms of clinically relevant depression, anxiety, insomnia, alcohol abuse and high stress by survey period (n = 3,052).

Variable		2022 (n = 1,031)	2024 (n = 2,021)	*P*
Depression	% (n)	28.3 (292)	21.6 (436)	<0.001
Anxiety	% (n)	16.2 (167)	14.8 (300)	0.33
Insomnia	% (n)	14.5 (150)	14.2 (286)	0.77
Alcohol Abuse	% (n)	17.9 (185)	20.9 (422)	0.055
High Stress	% (n)	56.3 (580)	51.0 (1031)	0.006

Mean symptom scores of depression, anxiety, and stress decreased from 2022 to 2024 (all p < 0.01) with small effect sizes (d from -0.17 to -0.11). No differences were found for mean insomnia and alcohol abuse scores (p = 0.90 and p = 0.052, respectively; **Table 2**).

**Table 2:** Mean scores of symptom scales for depression (PHQ-9), anxiety (GAD-7), insomnia (ISI-7), alcohol abuse (CAGE), and high stress (PSS-4) by survey period (n = 3,052).

Variable		2022 (n = 1,031)	2024 (n = 2,021)	*P*	*d*
PHQ-9	M (SD)	6.82 (5.62)	5.90 (5.29)	<0.001	-0.17
GAD-7	M (SD)	5.26 (4.53)	4.53 (4.62)	<0.001	-0.16
ISI-7	M (SD)	8.14 (6.01)	8.17 (5.60)	0.90	0.005
CAGE	M (SD)	0.688 (1.03)	0.766 (1.06)	0.052	0.074
PSS-4	M (SD)	6.02 (3.24)	5.64 (3.26)	0.002	-0.12

### 3.2 Sociodemographic correlates of clinically relevant mental health symptoms

Detailed results on the association of sociodemographic variables with mental health symptoms are summarized in **Supplementary Tables 2-6**. Only significant associations are reported in the following.

Female gender was associated with higher odds for symptoms of depression (aOR: 1.479 [1.170, 1.871]), anxiety (aOR: 1.412 [1.079, 1.848]), and stress (aOR: 1.362 [1.124, 1.650]), but lower odds for symptoms of alcohol misuse (aOR: 0.582 [0.463, 0.732]).

**Figure 1:**
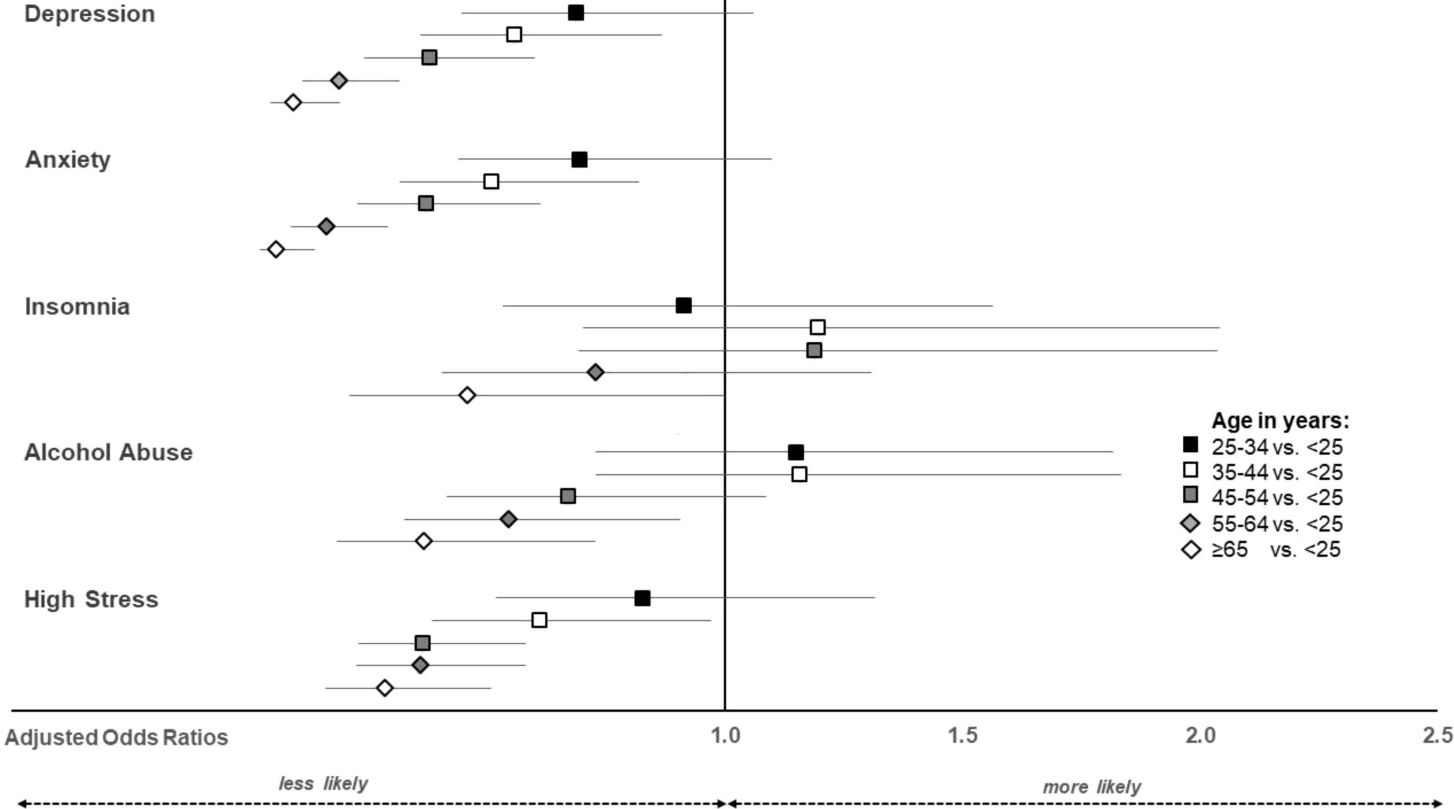
Adjusted odds ratios and their 95% confidence intervals for the odds of exceeding clinically relevant symptoms by age.

The odds of experiencing clinically relevant symptoms of depression, anxiety, alcohol abuse and high stress decreased with increasing age (**Figure 1**).

Migration background was associated with higher odds of depressive symptoms (aOR: 1.381 [1.010, 1.888]) and high stress (aOR: 1.770 [1.322, 2.369]) symptoms.

Being in a partnership vs. single reduced the odds for clinically relevant depressive symptoms (aOR: 0.706 [0.547, 0.911]).

**Figure 2:**
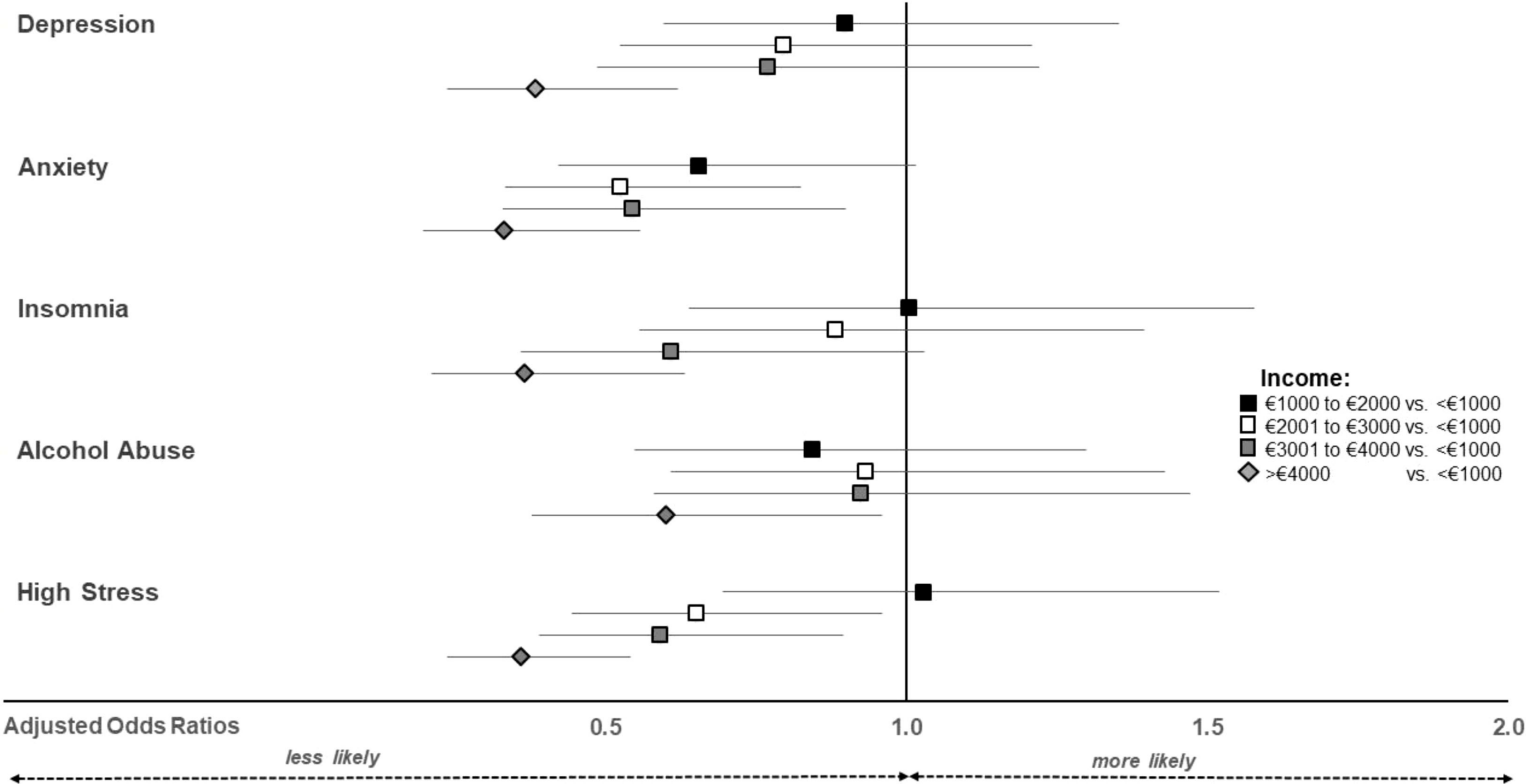
Adjusted odds ratios and their 95% confidence intervals for the odds of exceeding clinically relevant symptoms by net household income.

Participants of the highest income group (>€4,000 per month) had lower odds for all investigated mental health issues compared to those with an income of <€1,000 Euro (**Figure 2**). Further differences emerged for those with >€2000 vs. <€1,000 for reducing the odds for anxiety and high stress.

Being unemployed vs. employed increased the odds for depressive (aOR: 1.773 [1.260, 2.494]), anxiety (aOR: 1.641 [1.136, 2.371]), insomnia (aOR: 1.956 [1.344, 2.848]), and high stress symptoms (aOR: 1.575 [1.101, 2.253]).

Insomnia symptoms were less likely in participants with vocational secondary school education (aOR: 0.434 [0.263, 0.717]), high school education (aOR: 0.603 [0.375, 0.970]) or university education (aOR: 0.553 [0.326, 0.940]) vs. those with no formal or secondary education. Alcohol misuse symptoms were less likely in participants with apprenticeship (aOR: 0.596 [0.409, 0.870]) vs. those with no formal or secondary education. High stress symptoms were less likely in participants with vocational secondary school (aOR: 0.683 [0.466, 0.999]) and university education (aOR: 0.589 [0.392, 0.886]] vs. those with no formal or secondary education.

Regional differences revealed lower odds for depressive (aOR: 0.616 [0.411, 0.921]) and anxiety (aOR: 0.558 [0.347, 0.896] symptoms in participants from Styria vs. Vienna. Higher odds for symptoms of alcohol misuse were observed for inhabitants from Salzburg (aOR: 1.618 [1.014, 2.581]) and Burgenland (aOR: 2.152 [1.241, 3.730]) vs. Vienna. High stress symptoms were less likely in Upper Austria (aOR: 0.710 [0.518, 0.975] and Tyrol (aOR: 0.593 [0.401, 0.877] than in Vienna.

## 4 Discussion

### 4.1 Mental health indicators in October 2024 vs. April 2022

The observed decrease in depressive and stress symptoms among the Austrian population from April 2022 to October 2024 might be attributed to several interrelated factors that reflect changes in the social, economic, and psychological landscape during this period.

First, in April 2022, although the peak of the COVID-19 pandemic had passed, the pandemic was still a significant concern (7), and some measures were in place to manage ongoing risks (3). This environment likely contributed to lingering uncertainty and stress among the population. However, by October 2024, the pandemic had long since subsided, and public health measures were no longer in effect (3). The absence of restrictions and pandemic-related concerns in the media may have alleviated fear and stress. The Austrian general population described social contacts and recreational activities as the most helpful means of coping with stress during the pandemic (7). Returning to normalcy and engaging in social and recreational activities without the shadow of COVID-19 could have promoted better mental well-being.

Second, the stabilization of the economy and the return of inflation rates to normal levels (8, 23) might have reduced economic stressors that had previously contributed to mental health deterioration. In April 2022, survey responses indicated that inflation and financial concerns were the top stressors for the Austrian population, followed closely by the war in Ukraine (7). At that time, individuals faced the dual pressures of rising living costs and uncertainties regarding energy supplies exacerbated by geopolitical tensions. However, by October 2024, as inflation had returned to usual levels and real income levels increased (8, 23), individuals may have experienced improved financial security and reduced economic concerns. This stabilization could lead to a greater sense of control over one’s circumstances, thereby decreasing the prevalence of depressive and stress symptoms.

Moreover, increased awareness and access to mental health resources may have played a role in the decline of depressive and stress symptoms. As the public’s focus on mental health issues has intensified in recent years (1), particularly during and following the pandemic, individuals may have become more proactive in seeking help and utilizing available resources to decrease mental health burdens. Studies indicate that the pandemic led to a higher demand for psychotherapy (24) as well as an increase in the prescription of psychotropic medications (25) in Austria.

Further research is needed to explore the dynamics contributing to the decrease in mental health symptoms in depth, as understanding the factors that contribute to resilience and recovery can inform future mental health interventions and public health strategies. However, it is crucial to recognize that improvements were relatively small, and mental health has not returned to pre-pandemic levels.

### 4.2 Sociodemographic correlates of clinically relevant mental health outcomes

The association between female gender and younger age with a greater probability of experiencing mental health symptoms is supported by existing literature, which underscores that these demographic groups not only exhibit higher levels of clinically significant mental health issues but also endured more pronounced increases in mental distress throughout the pandemic (26).

The association between migration background and increased odds of depression and stress reflects the unique challenges faced by individuals from diverse backgrounds. Migrants may experience acculturative stress, discrimination, and economic hardships, which can exacerbate mental health issues (12). For refugees, these challenges are often compounded by pre-existing trauma from past experiences, which can accumulate over time in the host country (13). In general, cultural factors significantly shape mental health outcomes for migrants, acting as both protective and risk factors. Collectivist values may provide social support, but stigma around mental illness in some cultures can hinder help-seeking (27, 28). Language barriers, differences in health-seeking behaviors, and misalignment between traditional practices and Western approaches often exacerbate challenges (29). Religious and spiritual beliefs can offer resilience but may also create conflicts with host-country norms. Additionally, acculturative stress and identity struggles heighten vulnerability. Culturally sensitive mental health services, including interpreter support, diverse practitioners, and tailored interventions, are essential for addressing these disparities (30, 31).

Being in a partnership significantly reduced the odds of clinically relevant depressive symptoms, emphasizing the protective role of social support and emotional intimacy. Partnerships can provide individuals with resources for coping, which may mitigate the risk of developing mental health problems (17). Conversely, being single may increase feelings of loneliness and decrease the level of perceived social support (32), potentially leading to higher rates of depressive symptoms.

The results indicating lower odds of mental health issues among higher income groups highlight the critical role of socioeconomic status in mental health. Higher income often correlates with better access to healthcare, resources for coping with stress, and overall improved living conditions. In contrast, those with lower incomes may face additional stressors related to financial insecurity, limited access to healthcare, and a lack of supportive resources, contributing to increased vulnerability to mental health issues (14, 33).

Unemployment significantly increased the probability of depressive, anxiety, insomnia, and high-stress symptoms, reinforcing the link between economic stability and mental health. Employment provides not only financial security but also a sense of purpose and social interaction, both of which are vital for psychological well-being (34). The negative impact of unemployment on mental health is well-documented (35) and highlights the need for policies that support job creation and provide mental health resources for the unemployed. The mental health impact of unemployment is profound, but a more nuanced understanding can be achieved by considering the existing support systems in Austria. Austria’s labour market policies, which include both passive measures (such as unemployment benefits and assistance) and active measures (such as training programs, integration subsidies, and social enterprises), aim to address unemployment and its associated mental health burdens (36). These interventions provide crucial financial security and opportunities for re-entering the workforce, but their effectiveness in alleviating mental health issues might be further enhanced by integrating targeted mental health resources. Strengthening these services, particularly for long-term unemployed individuals, could mitigate the psychological impact of joblessness and improve overall well-being.

The findings related to educational attainment suggest that higher levels of education may confer protective benefits against specific mental health issues. This could be attributed to higher health literacy, personal resources, better coping mechanisms, and greater access to mental health resources among those with more education (14). Health literacy, in particular, may play a critical mediating role in these outcomes by enabling individuals to recognize symptoms, seek appropriate care, and adopt healthier behaviors. Future research should further investigate the pathways through which health literacy influences the relationship between educational attainment and mental health.

The findings indicate notable regional differences in mental health outcomes across Austria. Participants from Styria exhibited significantly lower odds of experiencing depressive and anxiety symptoms compared to those from Vienna, suggesting potential protective factors in this region, such as environmental, social, or economic conditions. In contrast, higher probability of alcohol misuse were observed in Salzburg and Burgenland compared to Vienna, which may reflect region-specific stressors or cultural factors contributing to alcohol consumption patterns. Regional differences in mental health outcomes may be influenced by varying social, economic, and environmental factors across Austria. These findings should be interpreted with caution, as acute crises such as the recent extreme weather events in Lower Austria and Vienna might temporarily inflate mental health symptoms and obscure underlying regional trends.

### 4.3 Limitations

The cross-sectional design limits the ability to make causal inferences about the relationship between sociodemographic factors and mental health outcomes. Longitudinal follow-up studies are needed to clarify these associations over time. Additionally, self-reported measures of mental health symptoms may be subject to response biases, such as social desirability or recall bias. These biases could influence the accuracy of the reported symptoms. Future research should consider employing mixed methods or incorporating objective measures where feasible to enhance the validity and generalizability of findings. The study also relied on online survey data, which may not fully represent individuals with limited internet access or lower digital literacy, potentially affecting the generalizability of the findings. This could result in underrepresentation of certain demographic groups, such as older adults, individuals living in rural areas, or those with lower incomes. Consequently, the findings may not fully generalize to the entire Austrian population. Future studies should consider employing mixed methods to enhance representativeness. Finally, the unique context of Austria may limit the generalizability to other regions or cultural settings.

### 4.4 Conclusions

This study points to the importance of ongoing mental health support in Austria. While the modest decrease in depressive and stress symptoms from April 2022 to October 2024 may reflect factors like the end of the pandemic, reduced inflation, and rising real incomes, mental health has not yet recovered to pre-pandemic levels. This suggests that the lingering impact of recent crises continues to affect the population’s overall well-being. Findings further highlight the complexity of mental health and the need to address sociodemographic disparities, which play a significant role in mental health outcomes. Policymakers should prioritize targeted interventions, such as increasing access to affordable mental health care, enhancing support systems for migrants, and addressing regional disparities through locally adapted strategies. Strengthening unemployment services with integrated mental health resources and promoting health literacy through educational initiatives could further mitigate these disparities.

## Data Availability

The raw data supporting the conclusions of this article will be made available by the authors, without undue reservation.
