# Supplementary material for: Mental health amidst multiple crises: trends and sociodemographic risk factors in Austria’s general population
Source: Front Psychiatry. 2025 Feb 27;16:1534994. doi: 10.3389/fpsyt.2025.1534994 (PMC11903441; doi:10.3389/fpsyt.2025.1534994)
Supplement: Supplementary file 1 [file Table1.docx]

Supplementary Material

**Suppl. Table 1.** Study sample characteristics (n = 2,025)

|  | N | % |
| --- | --- | --- |
| **Gender** |  |  |
| Male | 1025 | 50.6 |
| Female | 996 | 49.2 |
| Diverse | 4 | 0.2 |
| **Age** |  |  |
| 14-24 | 151 | 7.5 |
| 25-34 | 363 | 17.9 |
| 35-44 | 361 | 17.8 |
| 45-54 | 378 | 18.7 |
| 55-64 | 412 | 20.3 |
| ≥65 | 360 | 17.8 |
| **Region** |  |  |
| Vienna | 423 | 20.9 |
| Upper Austria | 325 | 16.0 |
| Lower Austria | 387 | 19.1 |
| Carinthia | 117 | 5.8 |
| Styria | 298 | 14.7 |
| Tyrol | 166 | 8.2 |
| Salzburg | 132 | 6.5 |
| Burgenland | 86 | 4.2 |
| Vorarlberg | 91 | 4.5 |
| **Education** |  |  |
| No school education | 3 | 0.1 |
| Secondary school | 199 | 9.8 |
| Apprenticeship | 780 | 38.5 |
| Vocational secondary school | 379 | 18.7 |
| High School | 365 | 18.0 |
| University | 299 | 14.8 |
| **Migration Background** |  |  |
| Yes | 269 | 13.3 |
| No | 1756 | 86.7 |
| **Work situation** |  |  |
| In employment | 1285 | 63.5 |
| Unemployed | 214 | 10.6 |
| Retired | 526 | 26.0 |
| **Net household income** |  |  |
| < € 1,000; | 176 | 8.7 |
| € 1,000,- to € 2,000,- | 472 | 23.3 |
| € 2,001,- to € 3,000,- | 546 | 27.0 |
| € 3,001,- to € 4,000,- | 351 | 17.3 |
| > € 4,000 | 480 | 23.7 |
| **Partnership status** |  |  |
| Single | 658 | 32.5 |
| Living in partnership | 1367 | 67.5 |

**Suppl. Table 2.** Results of the multivariable binary logistic regression analyses on the association of sociodemographic factors with the odds for symptoms of depression

|  | p-value | aOR | 95% CI | |
| --- | --- | --- | --- | --- |
| **Gender** (female vs. male) | .001 | 1.479 | 1.170 | 1.871 |
| **Age** |  |  |  |  |
| 25-34 years vs. <25 years | .089 | .690 | .449 | 1.059 |
| 35-44 years vs. <25 years | .009 | .559 | .361 | .867 |
| 45-54 years vs. <25 years | <.001 | .382 | .243 | .601 |
| 55-64 years vs. <25 years | <.001 | .190 | .114 | .317 |
| ≥65+ years vs. <25 years | <.001 | .095 | .047 | .190 |
| Migration background (yes vs. no) | .043 | 1.381 | 1.010 | 1.888 |
| **Education** |  |  |  |  |
| Apprenticeship vs. no education/secondary education | .696 | .926 | .632 | 1.358 |
| Vocational secondary school vs. no education/   secondary education | .230 | .762 | .489 | 1.188 |
| High school vs. no education/ secondary education | .393 | .830 | .541 | 1.273 |
| University vs. no education/secondary education | .423 | .826 | .517 | 1.320 |
| **Region** |  |  |  |  |
| Upper Austria vs. Vienna | .490 | .876 | .601 | 1.276 |
| Lower Austria vs. Vienna | .693 | 1.072 | .759 | 1.514 |
| Carinthia vs. Vienna | .153 | .649 | .358 | 1.175 |
| Styria vs. Vienna | <.001 | .616 | .411 | .921 |
| Tyrol vs. Vienna | .453 | .835 | .522 | 1.337 |
| Salzburg vs. Vienna | .959 | 1.013 | .624 | 1.644 |
| Burgenland vs. Vienna | .716 | 1.118 | .613 | 2.041 |
| Vorarlberg vs. Vienna | .054 | .520 | .268 | 1.010 |
| **Income** |  |  |  |  |
| € 1000,- to € 2000,- vs. < € 1000,- | .607 | .898 | .597 | 1.352 |
| € 2001,- to € 3000,- vs. < € 1000,- | .284 | .797 | .526 | 1.207 |
| € 3001,- to € 4000,- vs. < € 1000,- | .265 | .770 | .486 | 1.219 |
| > € 4000 vs. < € 1000,- | <.001 | .384 | .238 | .620 |
| **Partnerships status** (yes vs. no) | .007 | .706 | .547 | .911 |
| **Employment** |  |  |  |  |
| unemployed vs. employed | <.001 | 1.773 | 1.260 | 2.494 |
| retired vs. employed | .097 | 1.509 | .928 | 2.454 |

Note: Nagelkerke’s R-Square = 0.181. Grey shaded lines indicate significant results (p < .05).

**Suppl. Table 3.** Results of the multivariable binary logistic regression analyses on the association of sociodemographic factors with the odds for symptoms of anxiety

|  | p-value | aOR | 95% CI | |
| --- | --- | --- | --- | --- |
| **Gender** (female vs. male) | .012 | 1.412 | 1.079 | 1.848 |
| **Age** |  |  |  |  |
| 25-34 years vs. <25 years | .120 | .696 | .440 | 1.099 |
| 35-44 years vs. <25 years | .005 | .511 | .318 | .820 |
| 45-54 years vs. <25 years | <.001 | .373 | .228 | .613 |
| 55-64 years vs. <25 years | <.001 | .163 | .090 | .292 |
| ≥65+ years vs. <25 years | <.001 | .057 | .024 | .137 |
| **Migration background** (yes vs. no) | .736 | 1.065 | .740 | 1.531 |
| **Education** |  |  |  |  |
| Apprenticeship vs. no education/secondary education | .614 | .895 | .581 | 1.378 |
| Vocational secondary school vs. no education/   secondary education | .549 | .858 | .520 | 1.415 |
| High school vs. no education/ secondary education | .193 | .724 | .446 | 1.178 |
| University vs. no education/secondary education | .522 | .841 | .496 | 1.427 |
| **Region** |  |  |  |  |
| Upper Austria vs. Vienna | .829 | 1.047 | .690 | 1.588 |
| Lower Austria vs. Vienna | .982 | 1.005 | .677 | 1.490 |
| Carinthia vs. Vienna | .600 | .841 | .440 | 1.608 |
| Styria vs. Vienna | .016 | .558 | .347 | .896 |
| Tyrol vs. Vienna | .253 | .721 | .412 | 1.263 |
| Salzburg vs. Vienna | .807 | 1.070 | .621 | 1.846 |
| Burgenland vs. Vienna | .994 | 1.003 | .489 | 2.056 |
| Vorarlberg vs. Vienna | .083 | .490 | .219 | 1.097 |
| **Income** |  |  |  |  |
| € 1000,- to € 2000,- vs. < € 1000,- | .058 | .654 | .422 | 1.014 |
| € 2001,- to € 3000,- vs. < € 1000,- | .005 | .526 | .335 | .825 |
| € 3001,- to € 4000,- vs. < € 1000,- | .017 | .545 | .330 | .898 |
| > € 4000 vs. < € 1000,- | <.001 | .332 | .198 | .557 |
| Partnerships status (yes vs. no) | .308 | .859 | .641 | 1.151 |
| **Employment** |  |  |  |  |
| unemployed vs. employed | .008 | 1.641 | 1.136 | 2.371 |
| retired vs. employed | .125 | 1.563 | .884 | 2.766 |

Note: Nagelkerke’s R-Square = 0.165. Grey shaded lines indicate significant results (p < .05).

**Suppl. Table 4.** Results of the multivariable binary logistic regression analyses on the association of sociodemographic factors with the odds for symptoms of insomnia

|  | p-value | aOR | 95% CI | |
| --- | --- | --- | --- | --- |
| Gender (female vs. male) | .111 | 1.242 | .952 | 1.621 |
| Age |  |  |  |  |
| 25-34 years vs. <25 years | .742 | .914 | .534 | 1.563 |
| 35-44 years vs. <25 years | .506 | 1.198 | .703 | 2.041 |
| 45-54 years vs. <25 years | .529 | 1.189 | .694 | 2.035 |
| 55-64 years vs. <25 years | .291 | .730 | .407 | 1.309 |
| ≥65+ years vs. <25 years | .050 | .461 | .212 | 1.001 |
| Migration background (yes vs. no) | .086 | 1.366 | .957 | 1.950 |
| Education |  |  |  |  |
| Apprenticeship vs. no education/secondary education | .305 | .810 | .541 | 1.211 |
| Vocational secondary school vs. no education/  secondary education | .001 | .434 | .263 | .717 |
| High school vs. no education/ secondary education | .037 | .603 | .375 | .970 |
| University vs. no education/secondary education | .029 | .553 | .326 | .940 |
| Region |  |  |  |  |
| Upper Austria vs. Vienna | .832 | 1.047 | .683 | 1.606 |
| Lower Austria vs. Vienna | .445 | 1.166 | .786 | 1.731 |
| Carinthia vs. Vienna | .784 | .913 | .474 | 1.758 |
| Styria vs. Vienna | .495 | .856 | .547 | 1.338 |
| Tyrol vs. Vienna | .059 | .549 | .294 | 1.023 |
| Salzburg vs. Vienna | .510 | 1.203 | .695 | 2.083 |
| Burgenland vs. Vienna | .368 | 1.353 | .700 | 2.614 |
| Vorarlberg vs. Vienna | .433 | .747 | .361 | 1.548 |
| Income |  |  |  |  |
| € 1000,- to € 2000,- vs. < € 1000,- | .986 | 1.004 | .640 | 1.576 |
| € 2001,- to € 3000,- vs. < € 1000,- | .591 | .882 | .557 | 1.395 |
| € 3001,- to € 4000,- vs. < € 1000,- | .064 | .609 | .360 | 1.028 |
| > € 4000 vs. < € 1000,- | <.001 | .366 | .212 | .632 |
| Partnerships status (yes vs. no) | .351 | 1.151 | .857 | 1.545 |
| Employment |  |  |  |  |
| unemployed vs. employed | <.001 | 1.956 | 1.344 | 2.848 |
| retired vs. employed | .733 | 1.096 | .649 | 1.851 |

Note: Nagelkerke’s R-Square = 0.099. Grey shaded lines indicate significant results (p < .05).

**Suppl. Table 5.** Results of the multivariable binary logistic regression analyses on the association of sociodemographic factors with the odds for symptoms of alcohol abuse

|  | p-value | aOR | 95% CI | |
| --- | --- | --- | --- | --- |
| Gender (female vs. male) | <.001 | .582 | .463 | .732 |
| Age |  |  |  |  |
| 25-34 years vs. <25 years | .547 | 1.151 | .729 | 1.816 |
| 35-44 years vs. <25 years | .532 | 1.158 | .731 | 1.834 |
| 45-54 years vs. <25 years | .105 | .673 | .417 | 1.087 |
| 55-64 years vs. <25 years | .019 | .546 | .329 | .907 |
| ≥65+ years vs. <25 years | .004 | .369 | .187 | .728 |
| Migration background (yes vs. no) | .598 | .916 | .662 | 1.268 |
| Education |  |  |  |  |
| Apprenticeship vs. no education/secondary education | .007 | .596 | .409 | .870 |
| Vocational secondary school vs. no education/   secondary education | .130 | .721 | .472 | 1.102 |
| High school vs. no education/ secondary education | .088 | .698 | .462 | 1.055 |
| University vs. no education/secondary education | .107 | .694 | .445 | 1.083 |
| Region |  |  |  |  |
| Upper Austria vs. Vienna | .547 | 1.123 | .770 | 1.636 |
| Lower Austria vs. Vienna | .261 | 1.225 | .860 | 1.743 |
| Carinthia vs. Vienna | .860 | 1.050 | .612 | 1.800 |
| Styria vs. Vienna | .689 | .923 | .621 | 1.370 |
| Tyrol vs. Vienna | .868 | .961 | .599 | 1.541 |
| Salzburg vs. Vienna | .044 | 1.618 | 1.014 | 2.581 |
| Burgenland vs. Vienna | .006 | 2.152 | 1.241 | 3.730 |
| Vorarlberg vs. Vienna | .200 | 1.438 | .825 | 2.504 |
| Income |  |  |  |  |
| € 1000,- to € 2000,- vs. < € 1000,- | .440 | .844 | .550 | 1.298 |
| € 2001,- to € 3000,- vs. < € 1000,- | .748 | .933 | .609 | 1.429 |
| € 3001,- to € 4000,- vs. < € 1000,- | .741 | .925 | .582 | 1.470 |
| > € 4000 vs. < € 1000,- | .032 | .601 | .378 | .958 |
| Partnerships status (yes vs. no) | .415 | .899 | .696 | 1.162 |
| Employment |  |  |  |  |
| unemployed vs. employed | .919 | 1.020 | .703 | 1.478 |
| retired vs. employed | .931 | 1.021 | .638 | 1.636 |

Note: Nagelkerke’s R-Square = 0.074. Grey shaded lines indicate significant results (p < .05).

**Suppl. Table 6.** Results of the multivariable binary logistic regression analyses on the association of sociodemographic factors with the odds for symptoms of high stress

|  | p-value | aOR | 95% CI | |
| --- | --- | --- | --- | --- |
| Gender (female vs. male) | .002 | 1.362 | 1.124 | 1.650 |
| Age |  |  |  |  |
| 25-34 years vs. <25 years | .423 | .828 | .521 | 1.315 |
| 35-44 years vs. <25 years | .037 | .612 | .386 | .971 |
| 45-54 years vs. <25 years | <.001 | .367 | .232 | .582 |
| 55-64 years vs. <25 years | <.001 | .362 | .226 | .580 |
| ≥65+ years vs. <25 years | <.001 | .287 | .162 | .508 |
| Migration background (yes vs. no) | <.001 | 1.770 | 1.322 | 2.369 |
| Education |  |  |  |  |
| Apprenticeship vs. no education/secondary education | .134 | .767 | .542 | 1.086 |
| Vocational secondary school vs. no education/   secondary education | .049 | .683 | .466 | .999 |
| High school vs. no education/ secondary education | .154 | .755 | .513 | 1.111 |
| University vs. no education/secondary education | .011 | .589 | .392 | .886 |
| Region |  |  |  |  |
| Upper Austria vs. Vienna | .034 | .710 | .518 | .975 |
| Lower Austria vs. Vienna | .800 | .962 | .712 | 1.299 |
| Carinthia vs. Vienna | .072 | .664 | .425 | 1.037 |
| Styria vs. Vienna | .257 | .829 | .600 | 1.146 |
| Tyrol vs. Vienna | .009 | .593 | .401 | .877 |
| Salzburg vs. Vienna | .589 | .891 | .585 | 1.356 |
| Burgenland vs. Vienna | .732 | .916 | .555 | 1.511 |
| Vorarlberg vs. Vienna | .475 | .837 | .513 | 1.364 |
| Income |  |  |  |  |
| € 1000,- to € 2000,- vs. < € 1000,- | .894 | 1.027 | .695 | 1.518 |
| € 2001,- to € 3000,- vs. < € 1000,- | .029 | .652 | .444 | .958 |
| € 3001,- to € 4000,- vs. < € 1000,- | .013 | .591 | .391 | .895 |
| > € 4000 vs. < € 1000,- | <.001 | .360 | .239 | .541 |
| Partnerships status (yes vs. no) | .770 | 1.034 | .826 | 1.294 |
| Employment |  |  |  |  |
| unemployed vs. employed | .013 | 1.575 | 1.101 | 2.253 |
| retired vs. employed | .229 | .799 | .555 | 1.151 |

Note: Nagelkerke’s R-Square = 0.173. Grey shaded lines indicate significant results (p < .05).
